# Genetic association of NEAT1 gene polymorphism with the progression of colorectal cancer

**DOI:** 10.7150/jca.123558

**Published:** 2025-10-24

**Authors:** Bei-Hao Shiu, Yi-Hsien Hsieh, Chi-Chou Huang, Chih-Hsin Tang, Lun-Ching Chang, Shih-Chi Su, Shun-Fa Yang

**Affiliations:** 1 School of Medicine, Chung Shan Medical University, Taichung, Taiwan.; 2 Department of Surgery, Chung Shan Medical University Hospital, Taichung, Taiwan.; 3 Institute of Medicine, Chung Shan Medical University, Taichung, Taiwan.; 4 Department of Medical Research, Chung Shan Medical University Hospital, Taichung, Taiwan.; 5 Department of Pharmacology, School of Medicine, China Medical University, Taichung, Taiwan.; 6 Department of Medical Laboratory Science and Biotechnology, Asia University, Taichung, Taiwan.; 7 Chinese Medicine Research Center, China Medical University, Taichung, Taiwan.; 8 Department of Mathematics and Statistics, Florida Atlantic University, Florida, USA.; 9 Whole-Genome Research Core Laboratory of Human Diseases, Chang Gung Memorial Hospital, Keelung, Taiwan.; 10 Department of Medical Biotechnology and Laboratory Science, College of Medicine, Chang Gung University, Taoyuan, Taiwan.

**Keywords:** Colorectal cancer, nuclear enriched abundant transcript 1, single-nucleotide polymorphism, metastasis

## Abstract

Colorectal cancer (CRC) is a globally common malignancy, whose complex disease etiology involves a genetic element. *Nuclear enriched abundant transcript 1* (*NEAT1*), a long noncoding RNA (lncRNA) gene, has been demonstrated to play a key role in cancer development. To clarify the potential effect of *NEAT1* gene polymorphisms on CRC susceptibility, three *NEAT1* single-nucleotide polymorphisms (SNPs), including rs3825071, rs3741384, and rs512715, were assessed in 485 CRC patients and 485 sex- and age-matched non-cancer controls. We did not detect any significant association of these SNPs with the occurrence and clinicopathological features of CRC. Nevertheless, one SNP of *NEAT1* gene, rs3825071, was found in association with the distant metastasis (CT+TT: CC, OR, 2.644; 95% CI, 1.328-5.263; p=0.005) among relatively younger patients (< 65 years old), indicating an age-specific effect of *NEAT1* gene polymorphisms on the spread of CRC. Our stratification analysis revealed that the association of rs3825071 with CRC metastasis is anatomical site-specific, as cases of colon tumors but not of rectal tumors who bear at least one polymorphic allele of rs3825071 more commonly developed metastasis. Further exploration using the datasets from the Genotype-Tissue Expression (GTEx) Portal and The Cancer Genome Atlas (TCGA) showed that rs3825071 genotypes affected NEAT1 expression in the colon tissues, and elevated NEAT1 levels were associated with a worse survival rate in relatively younger patients (< 65 years old) with colon adenocarcinoma. These data suggest that altered expression levels of NEAT1 due to genetic polymorphisms may influence the progression of colon cancer.

## Introduction

Colorectal cancer (CRC) is one of the most common cancers globally 1. It remains the most prevalent malignancy among men and the second among women in Taiwan, representing a leading cause of deaths due to cancer 2. In spite of the recent progress on surgical procedures and other therapeutic strategies, the survival rate of CRC in Taiwan is lower than that in US 2, with the age-standardized death rate of CRC increasing over the years 3. Such high occurrence and mortality rates are mainly owing to the nature of its heterogeneous risk factors. Diet and long-lasting exposure of tumor-causing constituents (e.g., alcohol and cigarette) have been known as key environmental parameters that promote colorectal tumorigenesis 4. In addition, various genetic aberrations that affect proteolytic reactions, angiogenic responses, and cellular adhesion have been shown to orchestrate the development and progression of CRC 5. Recently, apart from host factors, disturbance in intestinal microbiota lying at the junction of those etiological parameters described above, has emerged as a major contributor of CRC 6. Taking the complexity of CRC pathogenesis into consideration, all these risks tend to be mutually interlinked and imperative to evaluate the disease prognosis.

Current advances on the exploration of long non-coding RNA (lncRNA) biology have led to a huge paradigm shift in functional genetics 7-9. To date, a great variety of lncRNAs are identified to play major roles in cancer 10, 11. One such lncRNA, nuclear enriched abundant transcript 1 (NEAT1), is bound by multiple RNA-interacting proteins to form nuclear paraspeckles, a group of highly dynamic nuclear subdomains that act as gene regulatory condensates in many healthy and disease settings, including malignancies 12. NEAT1 is functionally involved in tumorigenesis in many ways, comprising physical binding to microRNAs (miRNAs), orchestration of gene articulation, modulation of epigenetics, and engagement in signaling cascades 13. Moreover, the intricacy of NEAT1's functions in cancer development is amplified by its participation in regulating cancer cell stemness and metabolism 14. Recently, its functional association with autophagy further intensifies the complexity of NEAT1's roles in cancer biology 15. In CRC, NEAT1 is upregulated and induces colorectal cell carcinogenesis by targeting several miRNA/transcription factor axes, such as miR-34a/SIRT1/Wnt/β-catenin, miR-185-5p/IGF2, miR-495-3p/CDK6, miR-150-5p/CPSF4, and so on 16. Additionally, this RNA can interact with enhancer of zeste homolog 2 (EZH2), affecting the expression of genes involved in epithelial-to-mesenchymal transition (EMT) and tumor metastasis 17. Furthermore, serum NEAT1 levels are significantly elevated in patients with CRC compared to healthy controls 18. These findings suggest that NEAT1, functioning as a scaffold for RNA and protein molecules, is capable of governing the development and progression of CRC.

Lately, association studies using targeted gene approaches have unveiled a connection between single-nucleotide polymorphisms (SNPs) of NEAT1 gene and distinct types of cancers. Specifically, NEAT1 rs512715 was correlated with an increased risk of developing cervical cancer 19 and papillary thyroid carcinoma 20, as another SNP, rs2239895 polymorphisms, showed an association with the susceptibility to lung squamous cell carcinoma 21. In addition, a link of NEAT1 rs3825071 with the risk of gastric cancer 22 and clinical stage and lymph node metastasis of tongue cancer 23 was recently detected. As emerging roles of NEAT1 in CRC biology were demonstrated, the influence of NEAT1 gene polymorphisms on the development of CRC remains unexplored. Here, we conducted a hypothesis-driven survey to clarify a genetic association of NEAT1 SNPs with colorectal tumorigenesis.

## Materials and Methods

### Study cohort

This study was approved by the institutional review board of Chung Shan Medical University Hospital, Taichung (CSMUH No: CS1-20111), Taiwan and recruited 485 CRC cases and 485 cancer-free controls between 2016 and 2023 to explore the impact of NEAT1 gene variations on the development of CRC. Informed written consent was provided by each subject at enrollment. Staging and grading of tumors were evaluated by a pathologist using the American Joint Committee on Cancer (AJCC) TNM staging system 24. The control group encompassed subjects who did not report a history of cancer. Demographic data on age and gender were recorded from each participant.

### Genotyping

The three selected NEAT1 genetic variants (rs3825071, rs3741384, and rs512715) were chosen based on prior evidence linking them to cancer susceptibility 19-23. Specifically, rs3825071, rs3741384, and rs512715 have been associated with increased risks of cervical, thyroid, lung, gastric, and tongue cancers, highlighting their potential relevance to cancer development 19-23. A QIAamp DNA blood mini kit (Qiagen, Valencia, CA, USA) was used to isolate genomic DNA from whole blood samples 25, 26. Allelic discrimination for three NEAT1 SNPs was performed via the TaqMan assay with an ABI StepOne™ Real-Time PCR System (Applied Biosystems, Foster City, CA, USA).

### Statistical analysis

The differences in age and gender between cases and controls were assessed by using Fisher's exact test. The Hardy-Weinberg equilibrium was tested with a chi-square goodness-of-fit test for biallelic markers in both study cohorts. The adjusted odds ratios (AORs) with their 95% confidence intervals (CIs) were calculated by multiple logistic regression models with adjustment for age and gender for determining the association of NEAT1 genotypes with the risk of CRC. Difference in NEAT1 expression levels among genotypic groups from the Genotype-Tissue Expression (GTEx) database 27 was calculated with one-way ANOVA. The association of NEAT1 levels with the survival of patients from the colon adenocarcinoma dataset of The Cancer Genome Atlas (TCGA) was estimated with a Kaplan-Meier plotter and compared by using the log-rank test. Data were analyzed by using SAS statistical software (Version 9.1, 2005; SAS Institute Inc., Cary, NC). A p value < 0.05 was considered significant.

## Results

### Demographic and clinical characteristics of study cohorts

In this association study, we have recruited 485 CRC patients to examine whether variants of NEAT1 SNPs contribute to CRC susceptibility. Considering that chronological age and gender represent tentative risks for colorectal neoplasms 28, 485 cancer-free subjects with matched age and gender were recruited as the control group for comparisons. The demographic and clinicopathologic properties of the case and control group were analyzed (Table 1). Several anatomical sites were represented among the cases, including right-sided colon (30.3%), left-sided colon (46.8%), and rectum (22.9%). Histological examination confirmed advanced stage III/IV in 51.8% of cases. Lymph node and distant metastasis occurred in 49.9.5% and 15.9% of our cases, respectively.

### Age-specific effect of NEAT1 rs3825071 on CRC progression

To clarify whether NEAT1 gene polymorphisms are associated with the development and progression of CRC, rs3825071, rs3741384, and rs512715 were genotyped in this study. The genotypic frequencies of each SNP for both cohorts were examined (Table 2). Significant deviation from Hardy-Weinberg equilibrium in neither CRC nor control group was observed (p > 0.05) for three individual SNPs. Although none of these SNPs reached the threshold for significant associations, a marginal effect on CRC susceptibility was detected for specific genotypes of rs3741384 after the adjustment for potential confounders, age and gender (Table 2). Further assessment of NEAT1 association with clinicopathological characteristics of CRC patients also failed to detect any significant correlation of these NEAT1 variations with the progression of CRC (Table 3). Moreover, our stratification results demonstrated that the association of rs3825071 with distant metastasis of CRC (CT+TT: CC, metastatic tumor, OR, 2.644; 95% CI, 1.328-5.263; p=0.005) was exclusively detected in the younger age group (< 65 years old as the disease was diagnosed) (Table 4). Nevertheless, such genetic association with CRC metastasis was not seen in the more senior group (> 65 years old). These results indicate an age-specific effect of NEAT1 rs3825071 on the progression of CRC.

### rs3825071 was in association with metastasis of colon cancer but not that of rectal cancer

Given that rs3825071 was observed to be correlated with metastatic potential of CRC, we subsequently examined whether this genetic association was unique to the tumor location. We found that patients of colon cancer who carry at least one polymorphic allele of rs3825071 (CT and TT; OR, 1.919; 95% CI, 1.084-3.396; p = 0.024) more frequently developed distal metastasis (Table 5). Notably, this genetic association was exclusively observed in colon cancer but not in rectal cancer. These data indicate a promotive effect of NEAT1 gene polymorphisms on metastatic potential of colon adenocarcinoma but not on that of cancers in the rectum.

### Functional and clinical relevance of rs3825071 in CRC

**Figure 1:**
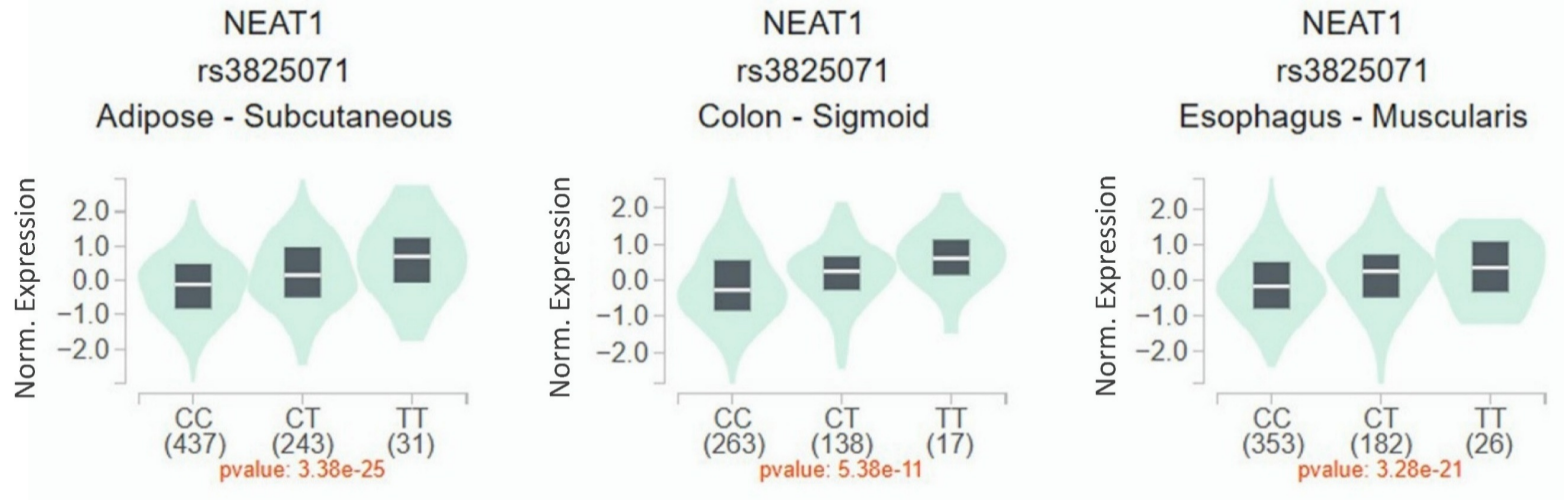
** rs3825071 regulates the expression of NEAT1.** eQTL analysis of rs3825071 in the adipose, colon tissues and esophagus tissue based on data from the GTEx portal. p value is calculated with one-way ANOVA.

**Figure 2:**
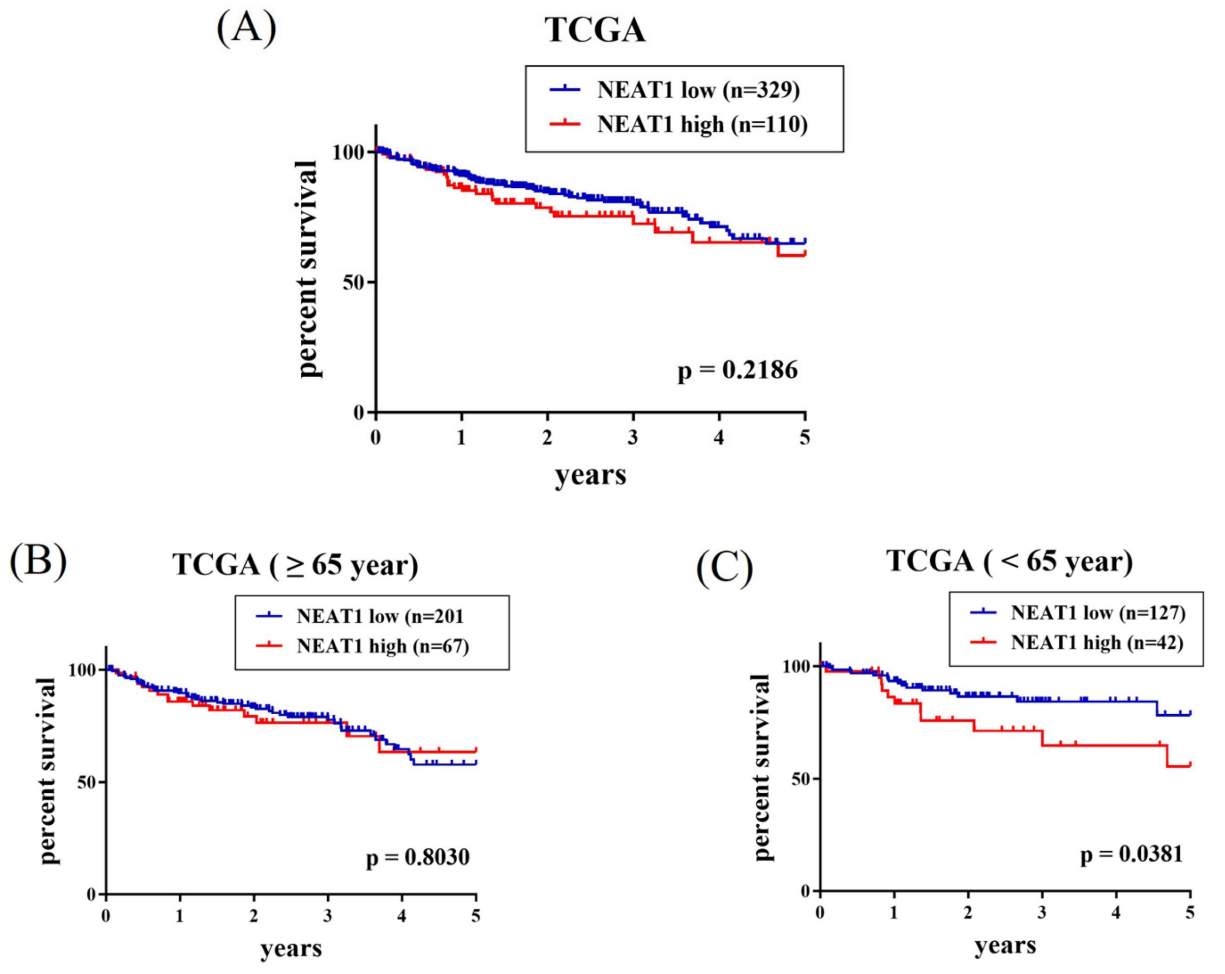
** Elevated NEAT1 expression levels are associated with a poor survival of patients with colon adenocarcinoma.** Survival analysis of patients with colon adenocarcinoma of (A) All cases group, (B) older age group (> 65 years old), and (C) younger age group (< 65 years old) from The Cancer Genome Atlas (TCGA) database based on NEAT1 expression. p values were analyzed by log-rank test.

Since a connection of rs3825071 with CRC metastasis was identified, extra analyses employing public datasets were conducted to gain putative functional insights of this CRC-associated SNP. We found alterations of NEAT1 expression in the adipose (p = 3.38×10-25), colon tissues (p = 5.38×10-11) and esophagus tissue (p = 3.28×10-21) among subjects who carry different rs3825071 genotypes in the Genotype-Tissue Expression (GTEx) database (Figure 1). Moreover, further analysis of data from patients with colon adenocarcinoma in TCGA dataset revealed that cases of the younger age group (< 65 years old) with tumors expressing higher levels of NEAT1 exhibited a worse survival rate than those with tumors expressing lower levels of NEAT1 (Figure 2A-2C). These results support genetic associations detected in our study and suggest that altered expression levels of NEAT1 due to genetic polymorphisms may affect the progression and prognosis of colon cancer in relatively younger patients.

## Discussion

The development and progression of colorectal tumorigenesis are a series of intricate processes modulated by the combination of both acquired and genetic parameters. In this survey, we reported that NEAT1 gene polymorphism, rs3825071, conferred the metastatic potential of CRC in an age- and anatomical site-specific manner. Furthermore, rs3825071 may be involved in the regulation of NEAT1 gene expression, which is associated with the survival in colon cancer patients of the younger age group.

Dysregulation of NEAT1 has been seen in various types of cancers, including CRC 16. In accordance with our data that rs3825071 genotypes affected NEAT1 expression in the colon tissues, gastric cancer patients carrying the minor allele of rs3825071 (CT and TT) were shown to express a higher level of NEAT1 in the whole blood specimens as compared with homozygotes for the major allele (CC) 22. These observations indicate a functional role of rs3825071 in acting as an expression quantitative trait locus (eQTL).

In addition to altered expression, functionality of rs3825071 might be also attuned based on the presence of polymorphic alleles. It has been predicted that the allelic change (C > T) of NEAT1 rs3825071 could give rise to corresponding alterations in the secondary structure of NEAT1 RNA transcripts, thereby abolishing the binding sites for hsa-miR-5092. Although the role of hsa-miR-5092 in CRC progression is yet mysterious, these findings collectively suggest that altered expression levels and sponging activities of NEAT1 rs3825071 attributed to its polymorphic alleles are likely implicated in governing the metastatic potential of CRC.

Moreover, it is striking that rs3825071 was merely linked to metastatic potentials of colon cancer whereas not to that of rectal cancer, indicating an anatomical site-specific influence of NEAT1 gene variations on the spread of colorectal neoplasms. Even though both rectal and colon malignancies arise in the large intestine and are usually viewed as a single tumor entity in most areas of clinical and basic research, considerable variations exist in molecular carcinogenesis, pathology, surgical topography and procedures, and multimodal treatment 29. In terms of the carcinogenic mechanism, colon tumors often displayed increased expression levels of HOX gene family 30, higher pathway activities of MAPK cascades 31, and constitutive activation of KRAS 32 and BRAF 33, 34, as compared to rectal tumors. Besides distinctive modulation of numerous cancer hallmarks in cancers of the rectum and colon, these interrupted carcinogenic signaling events collectively induced a key transcriptional activator of NEAT1, hypoxia-inducible factor 2α (HIF-2α) 35, resulting in the discrepancies of local NEAT1 expression within CRC tumor compartments. These evidence, to some degree, accords with our finding that rs3825071 was in association with CRC in an anatomical site-specific manner.

Another intriguing finding detected here is that association of NEAT1 rs3825071 with CRC metastasis was merely observed in younger patients, revealing a role of chronological age in the genetic susceptibility to CRC. In some conditions, genetic etiologies have been found to display a stronger explanatory power in younger individuals, as compared with the older group 36. This trend for genetic etiologies to lessen with increasing age has been reported in cancers 37, 38 and other conditions 39, 40. Nevertheless, the relevance of genetic risk factors to human diseases is not equal among age contexts, though the rationales behind this phenomenon remain unclear. Through employing a proportional hazards model within an interval-based censoring approach on datasets from the UK Biobank, several facets regarding the correlation of genetic relative risks with age were posed 36. Firstly, in a certain number of human disorders, statistical verification of a non-constant correlation between age and the impact of genetic etiologies was established. For such conditions, genetic etiologies mediated the largest influence at earlier ages, even though the magnitude and tendency of the drop-off varied across different diseases. Furthermore, the drop-off in genetic correlation with age cannot be attributed to latent variation in unmeasured covariates such as environmental parameters. These points of view underpin our observation that CRC is one of such illnesses affected by age-varying genetic risk profiles.

This investigation connected NEAT1 gene variants to the spread of colon cancer; however, additional efforts are necessary to deal with several study limitations. One caveat is the lack of data concerning the prolonged use of cigarettes and alcohol, potentially underestimating the impact of NEAT1 gene polymorphisms on the predisposition to CRC. Another issue is that the mechanistic role of rs3825071 in the promotion of colon cancer metastasis remains unanswered. How and to what extent the genetic polymorphism (C > T) affects its own expression or alters the interactions with its binding proteins or microRNAs needs further exploration. Moreover, in addition to rs3825071, another SNP (rs3741384) exhibited a marginal effect on CRC susceptibility. We acknowledge that many more significant associations are conceivably to be detected in a larger sample size. Lastly, the findings revealed in this study may be only applicable to particular ethnic groups and require replication experiments in different racial populations.

In conclusion, we detected a CRC-associated SNP in NEAT1 gene, rs3825071. Its connection with the metastatic responses of CRC was observed exclusively in younger patients (< 65 years old) with colon cancer. Induction of NEAT1 expression levels due to rs3825071 polymorphisms led to a poorer survival of younger patients with colon adenocarcinoma. These data unveil a novel link of NEAT1 variations to the progression and prognosis of CRC.

## Figures and Tables

**Table 1:** The distri butions of demographic and clinicopathologic characteristics in 485 controls and 485 patients with CRC.

Variable	Controls (N=485) n (%)	Patients (N=485) (%)	p value
**Age (yrs)**	60.09 ± 8.81	63.16 ± 13.08	
< 65	283 (58.4%)	255 (52.6%)	0.070
≥ 65	202 (41.6%)	230 (47.4%)	
**Gender**			
Male	297 (61.2%)	287 (59.2%)	0.512
Female	188 (38.8%)	198 (40.8%)	
**Tumor location**			
Rectum		111 (22.9%)	
Left colon		227 (46.8%)	
Right colon		147 (30.3%)	
**Stage**			
I+II		234 (48.2%)	
III+IV		251 (51.8%)	
**Tumor T status**			
T1-T2		120 (24.7%)	
T3-T4		365 (75.3%)	
**Lymph node status**			
N0		243 (50.1%)	
N1+N2		242 (49.9%)	
**Metastasis**			
M0		408 (84.1%)	
M1		77 (15.9%)	
**Lymphovascular invasion**			
No		272 (56.1%)	
Yes		213 (43.9%)	
**Perineural invasion**			
No		278 (57.3%)	
Yes		207 (42.7%)	
**Pathologic grading**			
Well		7 (1.4%)	
Moderately		441 (90.9%)	
Poorly		37 (7.7%)	

**Table 2:** Genotype distribution of *NEAT1* gene polymorphisms in 485 controls and 485 patients with CRC.

Variable	Controls (N=485) n (%)	Patients (N=485) n (%)	AOR (95% CI)	p value
**rs3825071**				
CC	336 (69.3%)	334 (68.9%)	1.000 (reference)	
CT	133 (27.4%)	142 (29.3%)	1.077 (0.813-1.427)	p=0.605
TT	16 (3.3%)	9 (1.8%)	0.600 (0.260-1.381)	p=0.230
CT+TT	149 (30.7%)	151 (31.1%)	1.027 (0.782-1.350)	p=0.846
**rs3741384**				
GG	350 (72.2%)	324 (66.8%)	1.000 (reference)	
GA	125 (25.8%)	151 (31.1%)	1.303 (0.983-1.728)	p=0.065
AA	10 (2.1%)	10 (2.1%)	1.099 (0.450-2.680)	p=0.836
GA+AA	135 (27.8%)	161 (33.2%)	1.288 (0.979-1.696)	p=0.071
**rs512715**				
GG	257 (53.0%)	254 (52.4%)	1.000 (reference)	
GC	186 (38.4%)	196 (40.4%)	1.063 (0.815-1.387)	p=0.651
CC	42 (8.6%)	35 (7.2%)	0.836 (0.516-1.354)	p=0.466
GC+CC	228 (47.0%)	231 (47.6%)	1.021 (0.793-1.315)	p=0.870

**Table 3:** Association between the clinical status and *NEAT1* genotypes in 485 CRC patients.

Variable	rs3825071			rs3741384			rs512715		
	CC (N=334)	CT + TT (N=151)	p value	GG (N=324)	GA + AA (N=161)	p value	GG (N=254)	GC + CC (N=231)	p value
Stages									
I+II	161 (48.2%)	73 (48.3%)	p=0.977	154 (47.5%)	80 (49.7%)	p=0.654	123 (48.4%)	111 (48.1%)	p=0.935
III+IV	173 (51.8%)	78 (51.7%)		170 (52.5%)	81 (50.3%)		131 (51.6%)	120 (51.9%)	
Tumor T status									
T1+T2	80 (24.0%)	40 (26.5%)	p=0.549	78 (24.1%)	42 (26.1%)	p=0.629	62 (24.4%)	58 (25.1%)	p=0.859
T3+T4	254 (76.0%)	111 (73.5%)		246 (75.9%)	119 (73.9%)		192 (75.6%)	173 (74.9%)	
Lymph node status									
Negative	168 (50.3%)	75 (49.7%)	p=0.898	163 (50.3%)	80 (49.7%)	p=0.898	130 (51.2%)	113 (48.9%)	p=0.619
Positive	166 (49.7%)	76 (50.3%)		161 (49.7%)	81 (50.3%)		124 (48.8%)	118 (51.1%)	
Metastasis									
Negative	288 (86.2%)	120 (79.5%)	p=0.059	271 (83.6%)	137 (85.1%)	p=0.680	217 (85.4%)	191 (82.7%)	p=0.408
Positive	46 (13.8%)	31 (20.5%)		53 (16.4%)	24 (14.9%)		37 (14.6%)	40 (17.3%)	
Lymphovascular invasion									
No	190 (56.9%)	82 (54.3%)	p=0.596	178 (54.9%)	94 (58.4%)	p=0.471	98 (54.1%)	98 (52.1%)	p=0.408
Yes	144 (43.1%)	69 (45.7%)		146 (45.1%)	67 (41.6%)		83 (45.9%)	90 (47.9%)	
Perineural invasion									
No	191 (57.2%)	87 (57.6%)	p=0.929	178 (54.9%)	100 (62.1%)	p=0.133	135 (53.1%)	137 (59.3%)	p=0.172
Yes	143 (42.8%)	64 (42.4%)		146 (45.1%)	61 (37.9%)		119 (46.9%)	94 (40.7%)	
Cell differentiation									
Well/ Moderately	306 (91.6%)	142 (94.0%)	p=0.352	301 (92.9%)	49 (91.3%)	p=0.533	139 (54.7%)	139 (60.2%)	p=0.226
Poorly	28 (8.4%)	9 (6.0%)		23 (7.1%)	14 (8.7%)		115 (45.3%)	92 (39.8%)	

**Table 4:** Association between the clinical status and *NEAT1* rs3825071 genotypes in 485 CRC patients of different age groups.

Variable	Aged < 65 (N=255)				Aged ≥ 65 (N=230)			
	CC (N=176)	CT + TT (N=79)	OR (95% CI)	p value	CC (N=158)	CT + TT (N=72)	OR (95% CI)	p value
Stages								
I+II	87 (49.4%)	38 (48.1%)	1.000	p=0.844	74 (46.8%)	35 (48.6%)	1.000	p=0.803
III+IV	89 (50.6%)	41 (51.9%)	1.055 (0.620-1.794)		84 (53.2%)	37 (51.4%)	0.931 (0.533-1.627)	
Tumor T status								
T1+T2	45 (25.6%)	22 (27.8%)	1.000	p=0.702	35 (22.2%)	18 (25.0%)	1.000	p=0.634
T3+T4	131 (74.4%)	57 (72.2%)	0.890 (0.490-1.617)		123 (77.8%)	54 (75.0%)	0.854 (0.445-1.639)	
Lymph node status								
Negative	90 (51.1%)	40 (50.6%)	1.000	p=0.941	78 (49.4%)	35 (48.6%)	1.000	p=0.915
Positive	86 (48.9%)	39 (49.4%)	1.020 (0.600-1.735)		80 (50.6%)	37 (51.4%)	1.031 (0.590-1.800)	
Metastasis								
Negative	156 (88.6%)	59 (74.7%)	1.000	p=0.005	132 (83.5%)	61 (84.7%)	1.000	p=0.822
Positive	20 (11.4%)	20 (25.3%)	2.644 (1.328-5.263)		26 (16.5%)	11 (15.3%)	0.916 (0.425-1.972)	
Lymphovascular invasion								
No	106 (60.2%)	43 (54.4%)	1.000	p=0.385	84 (53.2%)	39 (54.2%)	1.000	p=0.888
Yes	70 (39.8%)	36 (45.6%)	1.268 (0.742-2.167)		74 (46.8%)	33 (45.8%)	0.960 (0.549-1.680)	
Perineural invasion								
No	103 (58.5%)	46 (58.2%)	1.000	p=0.965	88 (55.7%)	41 (56.9%)	1.000	p=0.860
Yes	73 (41.5%)	33 (41.8%)	1.012 (0.591-1.734)		70 (44.3%)	31 (43.1%)	0.951 (0.542-1.668)	
Cell differentiation								
Well/ Moderately	165 (93.8%)	73 (92.4%)	1.000	p=0.691	141 (89.2%)	69 (95.8%)	1.000	p=0.100
Poorly	11 (6.2%)	6 (7.6%)	1.233 (0.439-3.461)		17 (10.8%)	3 (4.2%)	0.361 (0.102-1.272)	

**Table 5:** Association between the clinical status and *NEAT1* rs3825071 genotypes in 485 patients with tumors of different anatomical sites.

Variable	Rectum (N=111)				Colon (N=374)			
	CC (N=80)	CT + TT (N=31)	OR (95% CI)	p value	CC (N=254)	CT + TT (N=120)	OR (95% CI)	p value
Stages								
I+II	46 (57.5%)	15 (48.4%)	1.000	p=0.387	115 (45.3%)	58 (48.3%)	1.000	p=0.580
III+IV	34 (42.5%)	16 (51.6%)	1.443 (0.628-3.317)		139 (54.7%)	62 (51.7%)	0.884 (0.572-1.366)	
Tumor T status								
T1+T2	27 (33.8%)	10 (32.3%)	1.000	p=0.881	53 (20.9%)	30 (25.0%)	1.000	p=0.369
T3+T4	53 (66.2%)	21 (67.7%)	1.070 (0.442-2.590)		201 (79.1%)	90 (75.0%)	0.791 (0.474-1.320)	
Lymph node status								
Negative	47 (58.8%)	16 (51.6%)	1.000	p=0.496	121 (47.6%)	59 (49.2%)	1.000	p=0.782
Positive	33 (41.2%)	15 (48.4%)	1.335 (0.580-3.072)		133 (52.4%)	61 (50.8%)	0.941 (0.609-1.452)	
Metastasis								
Negative	66 (82.5%)	26 (83.9%)	1.000	p=0.863	222 (87.4%)	94 (78.3%)	1.000	p=0.024
Positive	14 (17.5%)	5 (16.1%)	0.907 (0.297-2.771)		32 (12.6%)	26 (21.7%)	1.919 (1.084-3.396)	
Lymphovascular invasion								
No	54 (67.5%)	18 (58.1%)	1.000	p=0.350	136 (53.5%)	64 (53.3%)	1.000	p=0.970
Yes	26 (32.5%)	13 (41.9%)	1.500 (0.639-3.520)		118 (46.5%)	56 (46.7%)	1.008 (0.653-1.558)	
Perineural invasion								
No	53 (66.2%)	21 (67.7%)	1.000	p=0.881	138 (54.3%)	66 (55.0%)	1.000	p=0.903
Yes	27 (33.8%)	10 (32.3%)	0.935 (0.386-2.263)		116 (45.7%)	54 (45.0%)	0.973 (0.629-1.506)	
Cell differentiation								
Well/ Moderately	80 (100.0%)	30 (96.8%)	1.000	p=0.107	226 (89.0%)	112 (93.3%)	1.000	p=0.182
Poorly	0 (0.0%)	1 (3.2%)	-----		28 (11.0%)	8 (6.7%)	0.577 (0.255-1.306)	
